# Data publication with the structural biology data grid supports live analysis

**DOI:** 10.1038/ncomms10882

**Published:** 2016-03-07

**Authors:** Peter A. Meyer, Stephanie Socias, Jason Key, Elizabeth Ransey, Emily C. Tjon, Alejandro Buschiazzo, Ming Lei, Chris Botka, James Withrow, David Neau, Kanagalaghatta Rajashankar, Karen S. Anderson, Richard H. Baxter, Stephen C. Blacklow, Titus J. Boggon, Alexandre M. J. J. Bonvin, Dominika Borek, Tom J. Brett, Amedeo Caflisch, Chung-I Chang, Walter J. Chazin, Kevin D. Corbett, Michael S. Cosgrove, Sean Crosson, Sirano Dhe-Paganon, Enrico Di Cera, Catherine L. Drennan, Michael J. Eck, Brandt F. Eichman, Qing R. Fan, Adrian R. Ferré-D'Amaré, J. Christopher Fromme, K. Christopher Garcia, Rachelle Gaudet, Peng Gong, Stephen C. Harrison, Ekaterina E. Heldwein, Zongchao Jia, Robert J. Keenan, Andrew C. Kruse, Marc Kvansakul, Jason S. McLellan, Yorgo Modis, Yunsun Nam, Zbyszek Otwinowski, Emil F. Pai, Pedro José Barbosa Pereira, Carlo Petosa, C. S. Raman, Tom A. Rapoport, Antonina Roll-Mecak, Michael K. Rosen, Gabby Rudenko, Joseph Schlessinger, Thomas U. Schwartz, Yousif Shamoo, Holger Sondermann, Yizhi J. Tao, Niraj H. Tolia, Oleg V. Tsodikov, Kenneth D. Westover, Hao Wu, Ian Foster, James S. Fraser, Filipe R. N C. Maia, Tamir Gonen, Tom Kirchhausen, Kay Diederichs, Mercè Crosas, Piotr Sliz

**Affiliations:** 1 Department of Biological Chemistry and Molecular Pharmacology, Boston, Massachusetts 02115, USA; 2 Laboratory of Molecular & Structural Microbiology, Institut Pasteur de Montevideo, Montevideo 11400, Uruguay; 3 Department of Structural Biology & Chemistry, Institut Pasteur, 75015 Paris, France; 4 Institute of Biochemistry and Cell Biology, Shanghai Institutes for Biological Sciences, Chinese Academy of Sciences, Shanghai 200031, China; 5 Harvard Medical School, Boston, Massachusetts 02115, USA; 6 NE-CAT and Department of Chemistry and Chemical Biology, Cornell University, Building 436E, Argonne National Laboratory, 9700S. Cass Avenue, Argonne, Illinois 60439, USA; 7 Departments of Pharmacology and Molecular Biophysics and Biochemistry, Yale University School of Medicine, New Haven, Connecticut 06520, USA; 8 Department of Chemistry, Molecular Biophysics and Biochemistry, Yale University, New Haven, Connecticut 06520, USA; 9 Bijvoet Center, Faculty of Science, Utrecht University, 3584 CH Utrecht, The Netherlands; 10 Departments of Biophysics and Biochemistry, University of Texas Southwestern Medical Center, Dallas, Texas 75390, USA; 11 Department of Internal Medicine, Washington University School of Medicine, St Louis, Missouri 63110, USA; 12 Department of Biochemistry, University of Zurich, CH-8057 Zurich, Switzerland; 13 Institute of Biological Chemistry, Academia Sinica, Taipei 11529, Taiwan; 14 Departments of Biochemistry and Chemistry, Center for Structural Biology, Vanderbilt University, Nashville, Tennessee 37232, USA; 15 Ludwig Institute for Cancer Research, San Diego Branch, La Jolla, California 92093, USA; 16 Department of Cellular and Molecular Medicine, University of California, San Diego, La Jolla, California 92093, USA; 17 Department of Biochemistry and Molecular Biology, SUNY Upstate Medical University, Syracuse, New York 13210, USA; 18 Department of Biochemistry and Molecular Biology, University of Chicago, Chicago, Illinois 60637, USA; 19 Department of Cancer Biology, Dana-Farber Cancer Institute, Boston, Massachusetts 02115, USA; 20 Edward A. Doisy Department of Biochemistry and Molecular Biology, Saint Louis University School of Medicine, St Louis, Missouri 63104, USA; 21 Departments of Chemistry and Biology and the Howard Hughes Medical Institute, Massachusetts Institute of Technology, Cambridge, Massachusetts 02139, USA; 22 Department of Biological Sciences and Center for Structural Biology, Vanderbilt University, Nashville, Tennessee 37235, USA; 23 Departments of Pharmacology and Pathology and Cell Biology, Columbia University, New York, New York 10032, USA; 24 Laboratory of RNA Biophysics, National Heart, Lung and Blood Institute, NIH, Bethesda, Maryland 20892, USA; 25 Department of Molecular Biology and Genetics, Weill Institute for Cell and Molecular Biology, Cornell University, Ithaca, New York 14853, USA; 26 Howard Hughes Medical Institute, Stanford University School of Medicine, Stanford, California 94305, USA; 27 Department of Molecular and Cellular Physiology, Stanford University School of Medicine, Stanford, California 94305, USA; 28 Department of Structural Biology, Stanford University School of Medicine, Stanford, California 94305, USA; 29 Department of Molecular and Cellular Biology, Harvard University, Cambridge, Massachusetts 02138, USA; 30 Key Laboratory of Special Pathogens and Biosafety, Wuhan Institute of Virology, Chinese Academy of Sciences, Wuhan 430071, China; 31 Howard Hughes Medical Institute, Harvard Medical School, Boston, Massachusetts 02115, USA; 32 Laboratory of Molecular Medicine, Boston Children's Hospital, Harvard Medical School, Boston, Massachusetts 02115, USA; 33 Department of Molecular Biology and Microbiology, Tufts University School of Medicine, Boston, Massachusetts 02111, USA; 34 Department of Biomedical and Molecular Sciences, Queen's University, Kingston, Ontario, Canada K7M 3G5; 35 Department of Biochemistry and Genetics, La Trobe University, Melbourne, Victoria 3086, Australia; 36 Department of Biochemistry, Geisel School of Medicine at Dartmouth, Hanover, New Hampshire 03755, USA; 37 Department of Medicine, University of Cambridge, MRC Laboratory of Molecular Biology, Francis Crick Avenue, Cambridge CB2 0QH, UK; 38 University of Texas, Southwestern Medical Center, Dallas, Texas 75390, USA; 39 Departments of Biochemistry, Medical Biophysics and Molecular Genetics, University of Toronto, Toronto, Ontario, Canada M5S 1A8; 40 Campbell Family Institute for Cancer Research, Ontario Cancer Institute/University Health Network, Toronto, Ontario, Canada M5G 2M9; 41 IBMC—Instituto de Biologia Molecular e Celular and Instituto de Investigação e Inovação em Saúde, Universidade do Porto, 4150 Porto, Portugal; 42 Université Grenoble Alpes/CNRS/CEA, Institut de Biologie Structurale, 38027 Grenoble, France; 43 Department of Pharmaceutical Sciences, University of Maryland, Baltimore, Maryland 21201, USA; 44 Howard Hughes Medical Institute and Harvard Medical School, Department of Cell Biology, Boston, Massachusetts 02115, USA; 45 Cell Biology and Biophysics Unit, Porter Neuroscience Research Center, National Institute of Neurological Disorders and Stroke, Bethesda, Maryland 20892, USA; 46 National Heart, Lung and Blood Institute, Bethesda, Maryland 20892, USA; 47 Department of Biophysics and Howard Hughes Medical Institute, University of Texas Southwestern Medical Center, Dallas, Texas 75390, USA; 48 Department of Pharmacology and Toxicology, Sealy Center for Structural Biology and Molecular Biophysics, University of Texas Medical Branch, Galveston, Texas 77555, USA; 49 Department of Pharmacology, Yale University School of Medicine, New Haven, Connecticut 06520, USA; 50 Department of Biology, Massachusetts Institute of Technology, Cambridge, Massachusetts 02139, USA; 51 Department of BioSciences, Rice University, Houston, Texas 77005, USA; 52 Department of Molecular Medicine, College of Veterinary Medicine, Cornell University, Ithaca, New York 14853, USA; 53 Department of Molecular Microbiology, Washington University School of Medicine, St Louis, Missouri 63110, USA; 54 Department of Pharmaceutical Sciences, College of Pharmacy, University of Kentucky, Lexington, Kentucky 40536, USA; 55 Departments of Biochemistry and Radiation Oncology, University of Texas, Southwestern Medical Center, Dallas, Texas 75390, USA; 56 Program in Cellular and Molecular Medicine, Boston Children's Hospital, Boston, Massachusetts 02115, USA; 57 Mathematics and Computer Science Division, Argonne National Laboratory, Argonne, Illinois, and Department of Computer Science, University of Chicago, Chicago, Illinois 60637, USA; 58 Department of Bioengineering and Therapeutic Sciences, University of California San Francisco, San Francisco, California 94158, USA; 59 Laboratory of Molecular Biophysics, Department of Cell and Molecular Biology, Uppsala University, Husargatan 3 (Box 596), SE-751 24 Uppsala, Sweden; 60 NERSC, Lawrence Berkeley National Laboratory, Berkeley, California 94720, USA; 61 Janelia Research Campus, Howard Hughes Medical Institute, Ashburn, Virginia 20147 USA; 62 Program in Cellular and Molecular Medicine and Department of Pediatrics, Boston Children's Hospital, Boston, Massachusetts 02115, USA; 63 Departments of Cell Biology, Harvard Medical School, Boston, Massachusetts 02115, USA; 64 Department of Biology, University of Konstanz, D-78457 Konstanz, Germany; 65 Institute for Quantitative Social Science, Harvard University, Cambridge, Massachusetts, 02138, USA

## Abstract

Access to experimental X-ray diffraction image data is fundamental for validation and reproduction of macromolecular models and indispensable for development of structural biology processing methods. Here, we established a diffraction data publication and dissemination system, Structural Biology Data Grid (SBDG; data.sbgrid.org), to preserve primary experimental data sets that support scientific publications. Data sets are accessible to researchers through a community driven data grid, which facilitates global data access. Our analysis of a pilot collection of crystallographic data sets demonstrates that the information archived by SBDG is sufficient to reprocess data to statistics that meet or exceed the quality of the original published structures. SBDG has extended its services to the entire community and is used to develop support for other types of biomedical data sets. It is anticipated that access to the experimental data sets will enhance the paradigm shift in the community towards a much more dynamic body of continuously improving data analysis.

## Abstract (web summary)

The validation and analysis of X-ray crystallographic data is essential for reproducibility and the development of crystallographic methods. Here, the authors describe a repository for crystallographic datasets and demonstrate some of the ways it could serve the crystallographic community.

## Graphical abstract

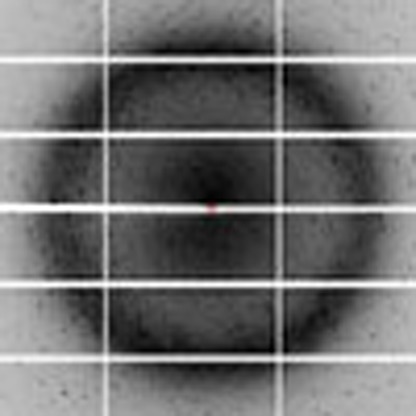

As one of the most powerful tools in structural biology, X-ray crystallography allows determination of the structure (atomic coordinates) of proteins, nucleic acids, small molecule compounds and macromolecular complexes to atomic-level resolution. Crystallographic data continue to be a primary source of mechanistic understanding of macromolecules, the implications of which extend from basic research to translational studies and the rational design of therapeutics. Reflecting the significance of the technique, the number of published macromolecular crystal structures has rapidly grown to >100,000 and numerous investigators within structural biology have been awarded the Nobel Prize, including Drs. Kendrew, Perutz, Watson, Crick, Wilkins, Hodgkin, Klug, Deisenhofer, Michel, Huber, Walker, MacKinnon, Kornberg, Ramakrishnan, Steitz, Yonath and Kobilka.

To support the needs of a growing structural biology community, a global network of synchrotron beamlines^1^ has been established and made available to researchers. These facilities remain the predominant source for crystallographic data collection. While the data collection process has become increasingly streamlined, deployment of a data management infrastructure to archive original diffraction images has been slow and uncertain^2^. With the exception of a modest number of data storage systems dedicated to the support of individual synchrotron beamlines^3^, or specific structural genomics projects^4^, storage of diffraction image data sets is typically the responsibility of primary investigators. Access to these original experimental data sets is therefore dependent on the policies of individual laboratories, which vary in storage organization, institutional resources, and researcher turnover. There is no universal archiving system to store X-ray diffraction data sets, and raw data sets are rarely made publicly available. In the cases where data sets are available, their distribution format can vary significantly. A typical data set of 360 images collected on modern detectors is 5 GB, and structure determination can involve one to tens of data sets, making the logistics of storing diffraction data for many protein structures a daunting task.

The benefits of easy and public access to experimental data are numerous^5^. Access to primary data would support community efforts to continuously improve existing models and identify new features through complete reprocessing of experimental data^6^,^7^,^8^ with modern software tools and improved criteria^9^. Further, original data may provide a basis for validating questionable existing structures while mistakes in structure determination may be identified earlier^10^,^11^,^12^. Additionally, access to a diverse volume of raw data can be used to develop improved software to address limitations of existing programs. Finally, access to a collection of varied experimental data will undoubtedly benefit the training and education of practitioners. The Worldwide Protein Data Bank^13^,^14^ (wwPDB) has illustrated how these achievements can be realized with the collection of reduced experimental data, in the form of structure factor amplitudes. Complementing this resource by preserving raw experimental data and making it available to a broad community promises a profound scientific impact in structural biology and other biomedical disciplines that face the challenges of preserving large data sets.

While the primary role of the SBGrid Consortium (www.sbgrid.org) has been to curate and support a collection of data processing software applications and to organize community-wide computing support^15^, SBGrid has also been active in the management of raw, experimental data sets. In 2012, SBGrid prototyped a system based on Globus technology^16^,^17^,^18^,^19^ to move diffraction data between Harvard, The Advanced Photon Source, and the Stanford Synchrotron Radiation Light source^19^.

To support the outstanding needs of the global structural community, we have established a publication system for experimental diffraction data sets that supports published structural coordinates: the Structural Biology Data Grid (SBDG). The SBDG project was initiated with a collection of X-ray diffraction image data sets as well as a few additional data set types contributed by many SBGrid Consortium laboratories. The collection supports a diverse subset of over 68 peer-reviewed publications and represents a sampling of numerous structure determination approaches. To evaluate the utility of such a data grid, we reprocessed all published diffraction data sets in this initial collection with modern software and compared the derived statistics against those reported in the original publications. We also demonstrate that by integrating the storage resources of multiple research groups and institutions, the data grid is poised to deliver a novel community driven data preservation system to support various types of structural biology and biomedical data sets.

## Results

### Structural biology data grid

**Figure 1:**
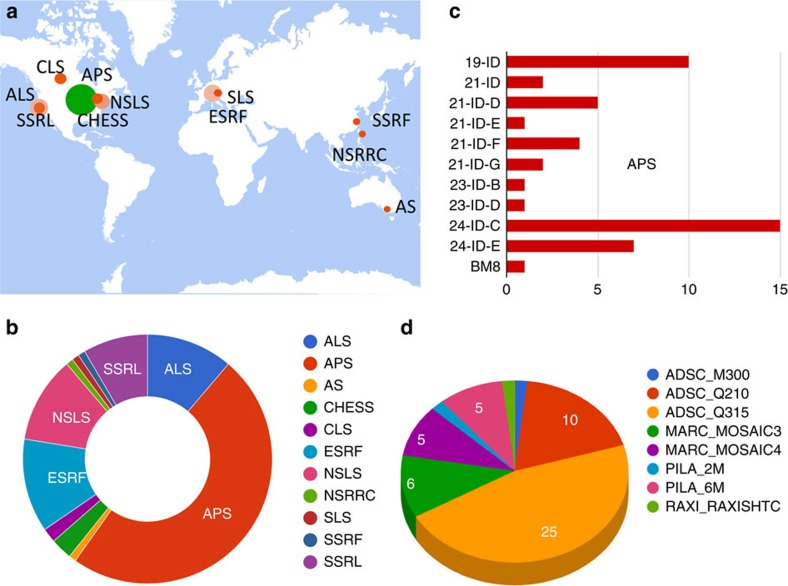
Data collection statistics for the pilot subset of 112 data sets. (**a**,**b**) Data sets were collected from synchrotrons on four continents (in addition to laboratory sources, which are not broken down geographically) and originate from eleven synchrotron facilities: Advanced Light Source, Advanced Photon Source, Australian Synchrotron, Cornell High Energy Synchrotron Source, Canadian Light Source, European Synchrotron Radiation Facility, National Synchrotron Light Source, National Synchrotron Radiation Research Center, Swiss Light Source, Shanghai Synchrotron Radiation Facility, and Stanford Synchrotron Radiation Lightsource. World map image courtesy of the U.S. Geological Survey. (**c**) Breakdown of data sets collected at the Advanced Photon Source beamlines. (**d**) Data sets cover a range of detector types, including Area Detector Systems Corporation M300, Q210 and Q315, Rayonix MarMosaic, Dectris Pilatus 2M and 6M, R-AXIS HTC, and MAR345.

**Figure 2:**
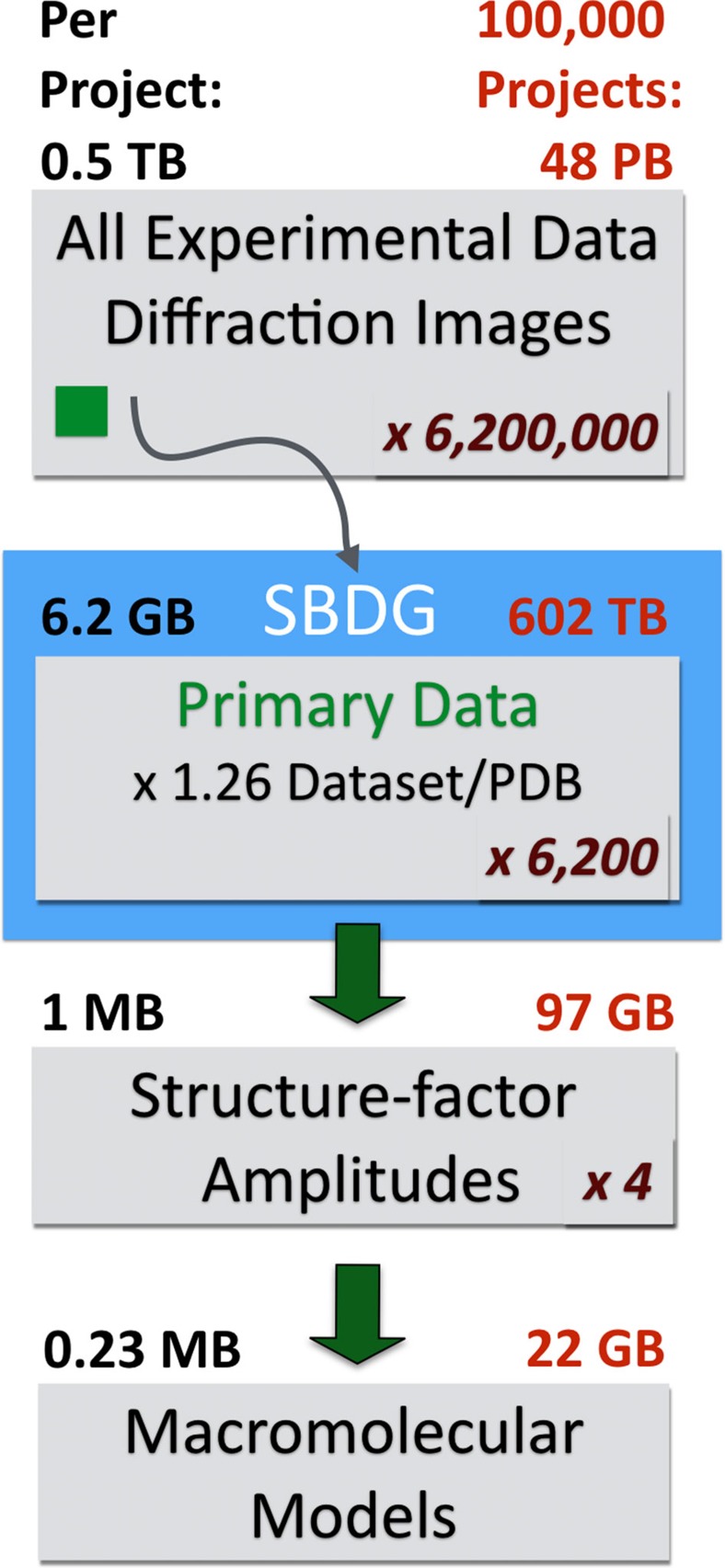
Estimation of storage requirements for different stages of the structural biology pipeline, based on the SBDG pilot collection. For structure factor amplitudes and PDB models file sizes were obtained from a subset of 96 PDB depositions derived from the pilot data sets. On average, SBDG stores 1.26 data sets per PDB file. Numbers in red indicated the estimated storage requirements to accommodate data sets for 100,000 structures. We estimate that for each primary data set, additional 100 data sets are collected at national facilities. Primary data refers to original experimental diffraction images supporting the derived structural model, as distinguished from all experimental data (screening images, inferior quality data sets, and so on). For crystallographic experiments, reduced data refers to the integrated intensities (or amplitudes, which do not materially affect storage requirements).

The SBDG is a centralized data publication service—a repository for discovering, downloading and depositing large structural biology data sets. We developed the SBDG to support the need of the SBGrid community to archive and disseminate X-ray diffraction image data sets, that is, images recorded on X-ray detectors, which support published structures. More than 90% of SBGrid laboratories use X-ray crystallography in their research, and SBGrid investigators have contributed over 11,000 X-ray structures to the PDB. The SBDG complements the PDB, which archives derived data—merged and post-refined data from diffraction images and the resulting refined coordinates of macromolecular structural models. The data grid has been developed in collaboration with the Data Science team at Harvard's Institute for Quantitative Social Science, and it conforms to progressive data science standards (Table 1). The SBDG limits its collection to data sets that support journal publications, referred to as ‘primary data'. For X-ray diffraction data, this primary data consists of experimental diffraction images supporting a derived structural model and journal publication. Release of this primary data by the SBDG coincides with publication of the resulting manuscript and for the structural biology data sets of related PDB files. As of 1 September 2015, the SBDG stores a diverse collection of 117 data sets, including 111 X-ray diffraction data sets and a handful of other data types including computational decoys and data sets from MicroED, lattice light-sheet microscopy and molecular dynamics (Supplementary Table 1). These published data sets, contributed by 50 laboratories with diffraction data sets collected at 11 synchrotron facilities (Fig. 1) and several home sources, originated 94 structures and 68 journal publications. The X-ray diffraction data sets range in size from 126 MB (ref. 20) to 20 GB (ref. 21) with a mean of 4.9 GB and a total of 573 GB of storage. Extrapolating from this initial collection, which is quite diverse and registers at just over 0.5 TB, our current 100 TB file system could immediately support roughly twenty thousand X-ray diffraction data sets (Fig. 2).

**Figure 3:**
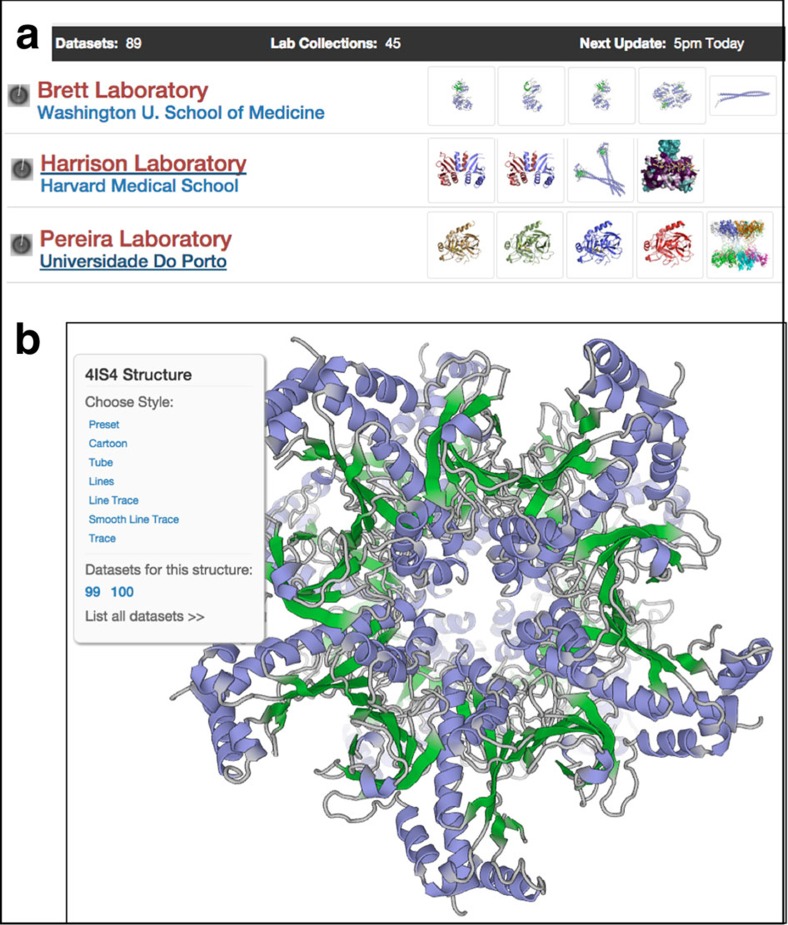
Organized display of data collections at SBDG. (**a**) Graphical view of Laboratory and Institutional Collections within the SBDG; (**b**) PV structure viewer, displaying a published model with links to its two primary deposited data sets.

The SBDG's collection of data sets can be accessed from the data.sbgrid.org website. On the home page, deposited data sets are organized into laboratory and institutional collections (Fig. 3a). Hyperlinked collection pages provide a list of selected data sets along with the data set's corresponding data Digital Object Identifier (DOI), a link to the journal publication, the PDB ID, a link to the PDB entry, and a link to the depositors' laboratory website. The website molecular viewer, PV^22^, offers visitors an option to view structures in a manipulatable cartoon representation (Fig. 3b). With multiple high-quality viewing options and flexible search functionality, users of the SBDG website can easily identify a small subset of relevant data sets.

**Figure 4:**
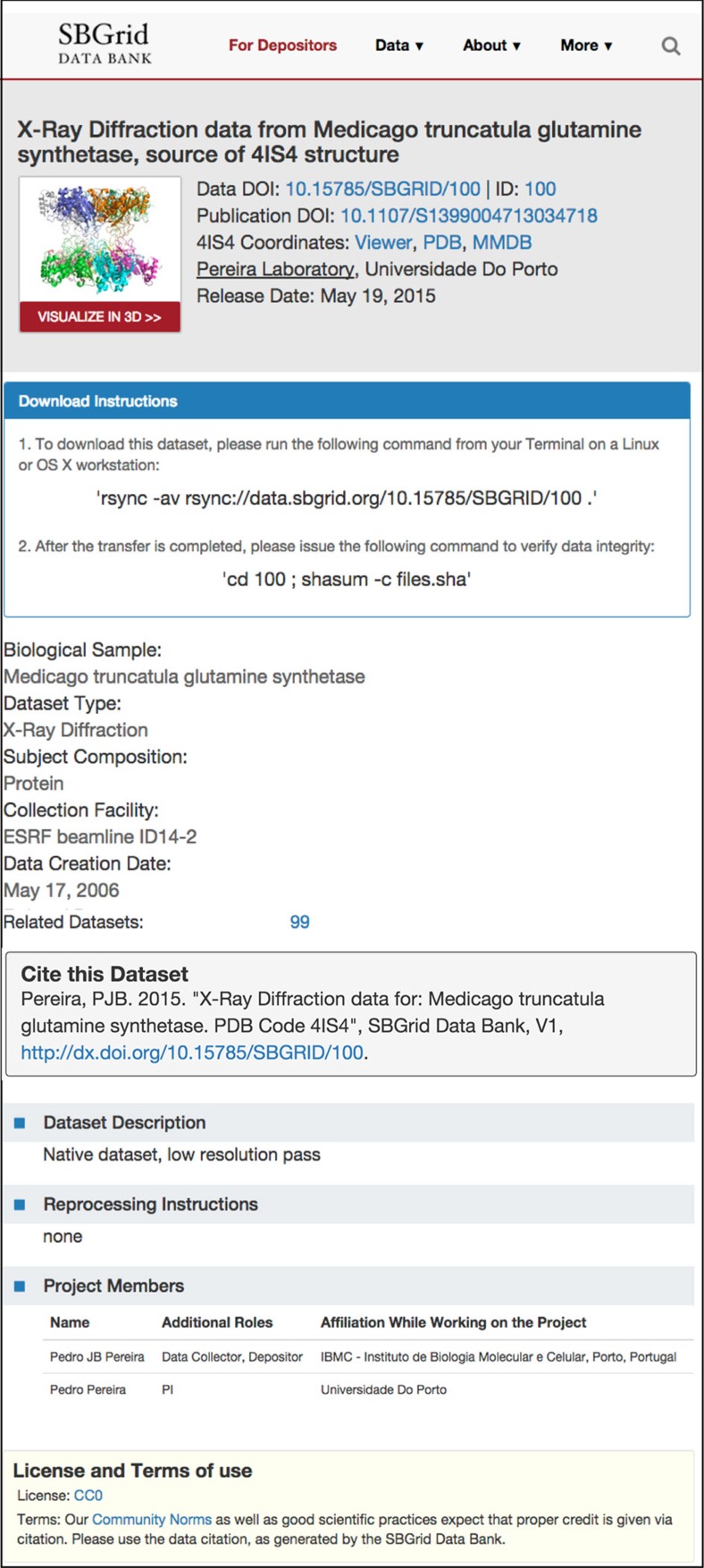
SBDG persistent data set landing page (the target of a DOI resolver for a published data set). Data set metadata are displayed, as are instructions for downloading and verifying the data set.

Persistent data set pages are an important element for any research data repository because they typically provide a landing URL, which resolves from a given DOI^23^. The SBDG does not advertise unique codes, but instead distinguishes data sets by fully qualified DOIs. From each SBDG collection page or viewer page a user can access those unique Data set Pages (Fig. 4), which offer additional information for each data set including download instructions and the fully formatted data set citation for inclusion in manuscripts, following best practices set by the Joint Declaration of Data Citation Principles^24^. A Data set Page can also be located by searching the SBDG for a PDB code, although often several related data sets are used to determine a single set of macromolecular coordinates. As the Data Grid is developed, the Data set Pages will include additional functionality, with more information on how to reprocess data sets, extended data statistics, and discussion forums allowing users to annotate data sets after publication. Taken together, the uniquely defined Data set Pages provide a comprehensive and persistent location for individual data sets.

### Data set access

All data sets in the SBDG are readily and freely accessible to the community. Access rights were formalized with adoption of the creative commons zero licence (CC0), which supports dedication of research results to the public domain and is used by many open-data projects. This licence allows use and redistribution of data for both commercial and non-commercial purposes without requiring additional agreements. The CC0 licence does not affect patents or trademark rights of contributors, and is similar to the licensing terms that are used for macromolecular models released by the wwPDB.

**Figure 5:**
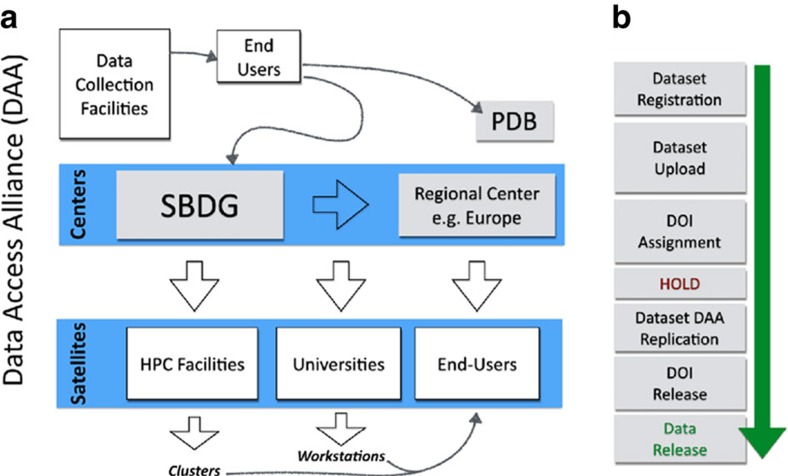
Experimental data flow and publication. (**a**) Flow of Primary Experimental Data. Data sets collected at synchrotrons are moved to end-users' computers for processing and structure determination. Subsequently refined macromolecular models are deposited at PDB and primary data is uploaded to SBDG. From SBDG, data sets are replicated to DAA centres and eventually copied to DAA Satellites. End-users can access data sets by downloading from DAA centres and by direct access from Satellites. (**b**) Flowchart for data publication.

Although data sets can be downloaded individually, their size can make this cumbersome. Physical access to SBDG data sets is facilitated through a data grid infrastructure that is supported by members of the data access alliance (DAA; Fig. 5a). The DAA is a voluntary and open organization of research-data-storage providers and is being developed in collaboration with the Globus Project. The DAA has two aims: (1) to minimize the chance of data loss by replicating SBDG data sets, and (2) to facilitate global data access through its members. Although it is expected that DAA membership and architecture will evolve rapidly, in its current state the DAA framework already provides a global solution for data dissemination. DAA centres in Europe, Asia, North America and South America replicate the entire SBDG collection and provide local access to members of regional communities. There are four DAA centres: Harvard Medical School in the USA, Uppsala University in Sweden, Shanghai Institutes for Biological Sciences in China, and Institut Pasteur de Montevideo in Uruguay. As a secondary service, DAA centres can provide local, direct access to data sets for their institutional research groups. For example, Harvard Medical School hosts the entire collection and provides direct access to all data from its computing center. The DAA infrastructure is further extended by the DAA satellites, which replicate fractions of SBDG data sets in their local storage for direct access by members of individual institutions. This mode of participation provides an attractive option for research institutions to develop local archives of all primary data generated by the local community. For example, the NE-CAT (Northeastern Collaborative Access Team; sector 24-ID) synchrotron beamline at the Advanced Photon Source, in Argonne, IL, replicates all SBDG data sets that originate from NE-CAT beamlines and makes them available to beamline staff and users. Another SBGrid member and DAA Satellite, Yale University, replicates all data sets from Yale laboratories on its institutional storage and makes them accessible to structural biology workstations through the Network File System. We expect that, as research storage infrastructure catches up with the capacities required to archive larger collections of diffraction data sets, some DAA satellites will elect to replicate a larger fraction of SBDG archives and make them available to the general community.

While the DAA offers a variety of data access options that will support growth of the repository, members of the community can also download individual data sets directly from SBGrid servers at Harvard using an rsync protocol. Instructions for downloading individual data sets are provided on the Data set View Pages, and effectively all data sets can be downloaded using the following command: ‘rsync -av rsync://data.sbgrid.org/DOI.', where DOI is the digital object identifier for a particular data set. The rsync utility, which is native to Linux and OS X systems, is particularly suitable for downloading large data files and can be restarted in case of interruption. After download, the data integrity of individual data sets can be verified by following instructions on the Data Grid website. With a well defined and permissive CC0 access licence and multiple channels for accessing data (four DAA sites and the rsync download mechanism) our initial infrastructure is well suited to support expansion of the data collection.

### Data publication cycle

For many SBGrid laboratories, interest in data deposition is driven by a desire to better organize research data and comply with institutional, federal, and project-specific data preservation requirements. During the pilot phase, data deposition privileges were limited to SBGrid member laboratories. With recent funding to further support the project, the Data Grid is now open to the entire structural biology community. Non-SBGrid groups would first need to register with the SBDG to obtain proper deposition credentials.

**Figure 6:**
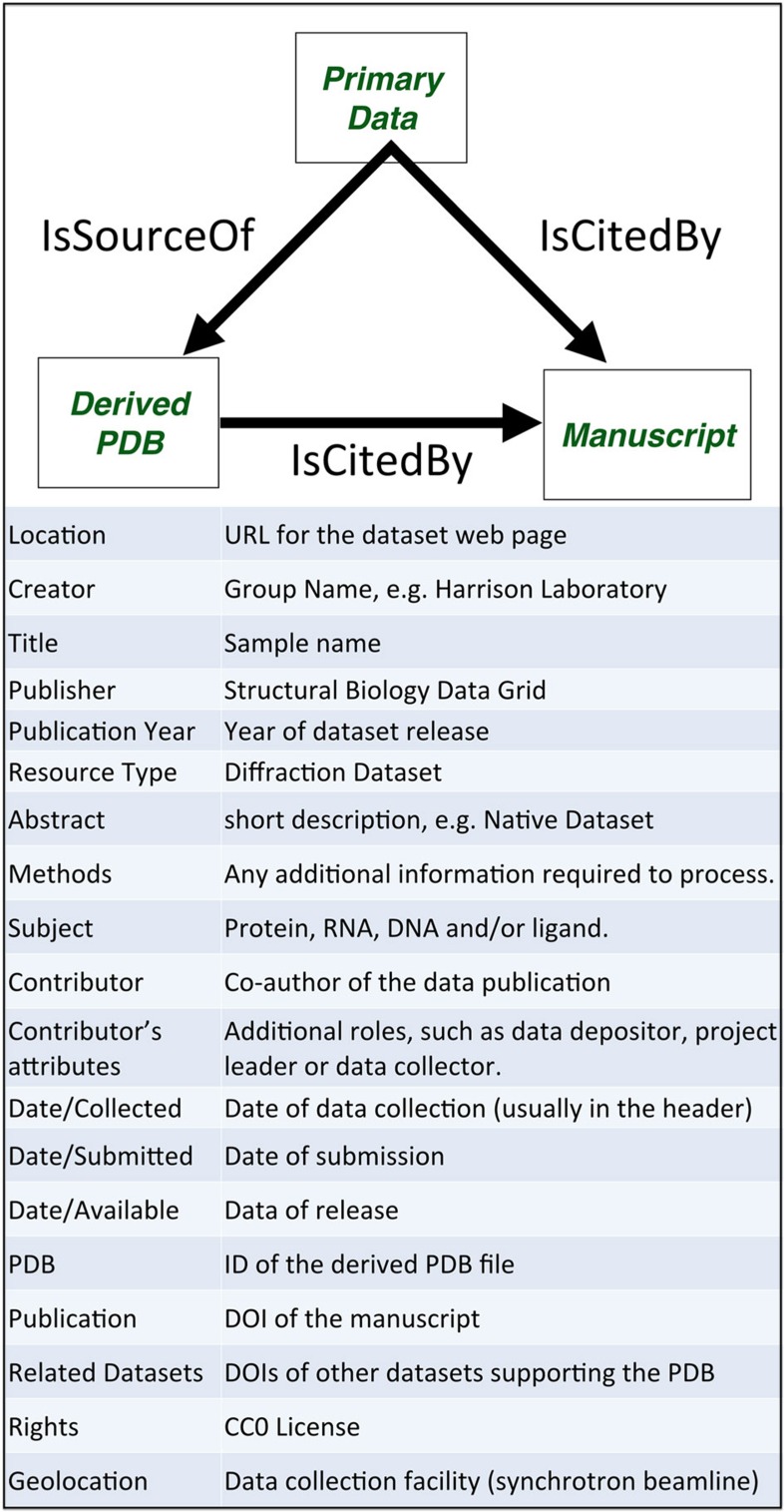
DataCite metadata schema used for primary data sets within the SBDG. Information associated with the DOI record for a primary data set through the EZID system.

Wide adoption of data preservation systems is often hindered by the complexities involved in the data deposition process itself. To mitigate this problem, SBDG deposition involves two simple steps: registration and uploading (Fig. 5b). To register a data set, the depositor completes a web form with basic information about the sample, data collection facility, related objects (for example, publication, PDB code), and authorship; this information is mapped to the DataCite schema (Fig. 6). Many details necessary for data set reprocessing—beam center, distance, wavelength, and so on—are automatically included with most data sets in the form of an image header generated by the data collection software at the time of collection, simplifying the registration process. A principal investigator is authorized to sponsor depositions as a recognized member of the community and must approve each deposit. This system allows maximum flexibility when accepting data for deposition, facilitating the upload of complex data sets that otherwise could be challenging to validate. Following registration, a DOI is reserved for the data set and the user is provided with data transfer instructions. Data deposition is handled by an automated script provided by SBDG and run on the depositor's computer, which uploads the data and checks for data integrity after upload. Upon verification, the primary data are either released in the bi-weekly SBDG release or placed on hold. As with the PDB, release of data placed on hold will coincide with publication.

The two-step publication process is complemented by behind-the-scenes data replication, DOI registrations, and data analysis. All X-ray diffraction images are currently post-processed using data processing pipelines that provide a post-publication data review that will be shared with depositors and the community in the next phase of the SBDG project. We are building additional tools to help increase data deposition rates, including automatic reminders sent to consortium members to encourage them to deposit data for previously published work.

### Data citation

**Figure 7:**
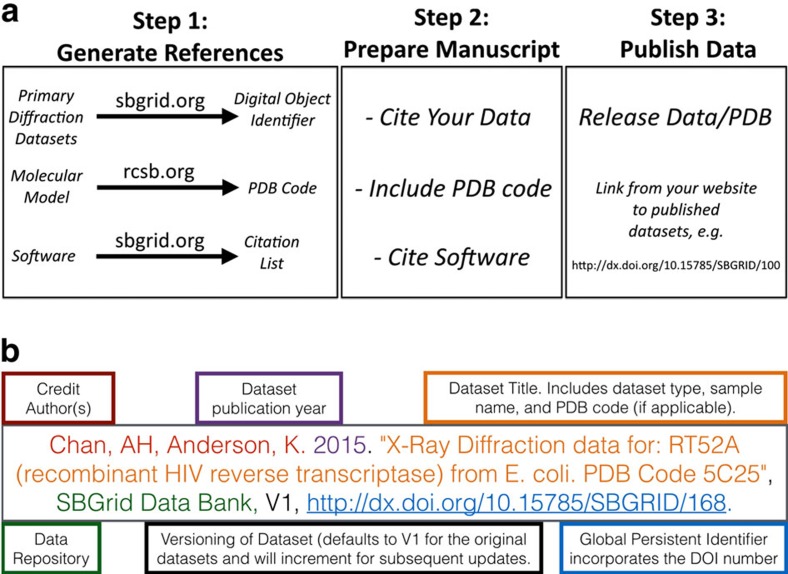
Data publication guidelines. (**a**) Flowchart illustrating publication guidelines incorporating software and data citations. (**b**) Data Citation guidelines, adapted from Dataverse Best Practices Guidelines that were developed based on Force 11 Joint Declaration of Data Citation Principles.

Research data are the legitimate and citable product of research^24^,^25^ and, therefore, the SBDG recommends that depositors and data users cite all data deposited with the SBDG in the standard reference section of their manuscripts following well established community standards^24^,^26^,^27^. Data citation examples are provided on individual data set pages (Fig. 4). The SBDG complements our AppCiter application^28^, which facilitates citation of research software. Both services are now presented to users in a unified publication support workflow (Fig. 7a). In step 1, the user deposits research-related data that are put on hold until publication. A set of DOIs and corresponding data citations are then generated and provided to the end-user. Users can also use AppCiter to generate a list of software citations for all scientific software used in the project. In step 2, all research data and scientific software citations are included in the References section of the manuscript. In step 3 the user, anticipating manuscript publication, contacts relevant databases to request release of the primary and supporting data. This process should, ideally, take place before manuscript publication and be timed to coincide with the publication date, allowing the community to access the data when the manuscript is released. When preparing future publications that refer to completed structures, scientists should reference the relevant publications and macromolecular models, unless they are referring to a specific data set. For specific data sets, authors should explicitly reference experimental data using the corresponding data citation (Fig. 7b). Citation metrics for published data sets will be comparable to those obtained for journal publications.

### Data grid content

Ease of data deposition and community-wide interest facilitated growth of the initial collection of X-ray diffraction data sets when it opened to the SBGrid community in May 2015. The data sets deposited during the pilot collection phase represent a wide cross-section of structures and a diverse subset of journal articles and structure determination methods. For example, 68 structures derived from data deposited in the SBDG have been determined by molecular replacement, while 4 have been solved by Multiple-wavelength Anomalous Diffraction, 4 by Single Isomorphous Replacement with Anomalous Scattering and 15 by Single-wavelength Anomalous Diffraction. The highest resolution data set^29^ extended to 1.04 Å, and the lowest resolution data set^30^ to 5.5 Å. The structures ranged in molecular weight from 8.1 (ref. 31) to 426 kDa (ref. 32). The solvent content of these structures ranged from 32 (ref. 33) to 85% (ref. 34) and the longest unit cell edge was reported to be 525.29 Å (ref. 35).

**Figure 8:**
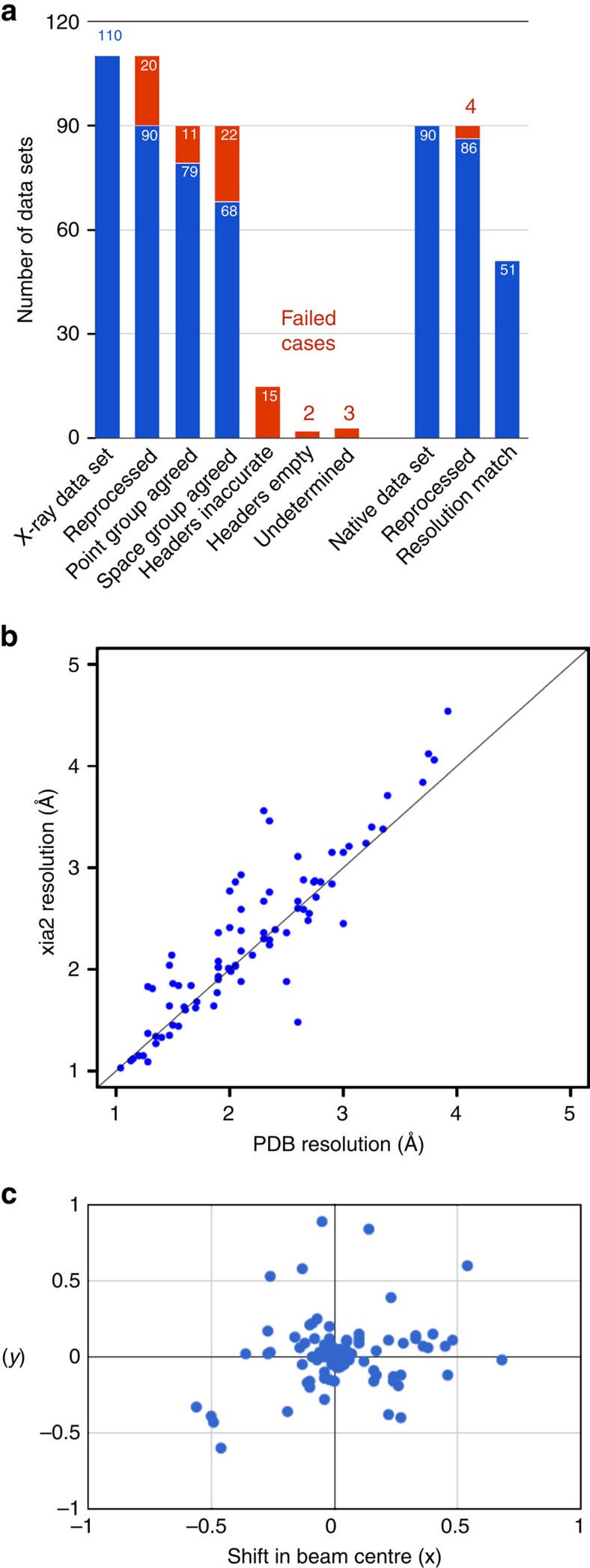
Reprocessing of X-ray diffraction data sets. (**a**) Analysis of 110 X-ray diffraction data sets that supported previously published PDB coordinates. Most of the failures (represented in red) were due to inaccurate or incomplete image-header information. In several of these cases, depositors provided annotations correcting this information; (**b**) Comparison of resolution determined by automated *xia2* reprocessing with published resolution. Includes data sets not used for final refinement of published structures; (**c**) Shift in direct beam position from image headers and refined value following successful reprocessing with *xia2*.

For a proof of concept, released data sets in the SBGridDB were reprocessed with *xia2* (refs 36, 37, 38, 39, 40, 41, 42) in a fully automated manner (Fig. 8a). In all, 90 of the 110 released data sets with a corresponding PDB ID were successfully reprocessed. In all,86 of those 90 data sets represented high-resolution, native data and for 51 of those *xia2* decision making determined a high-resolution limit within 0.1 Å of the published structure (Fig. 8b). The point group determined by reprocessing agreed with that of the published structure in 79 cases; for 65 of these the space groups agreed. The lower degree of recovery of space groups, in comparison to point groups, is attributed to ambiguity in screw axis determination at this stage of data processing. To provide insight into the most common failure modes, data sets for which *xia2* did not produce a set of integrated intensities were investigated using iMOSFLM^43^. Twelve of the failure cases could be attributed to absent or inaccurate information in the image headers: while accuracy of the beam center annotation varied within the pilot collection (Fig. 8c), 10 data sets had visually incorrect beam center information, two had missing header information. The cause of failure for the eight remaining data sets was not definitively determined from the data sets alone; however, consulting the reprocessing instructions provided by depositors clarified this for five of these data sets. The reprocessing instructions also suggested that many of the data sets for which *xia2* was able to produce integrated intensities, but with resolution or symmetry disagreeing with the deposited structure, could be attributed to incorrect header information. One outlying reprocessing case for which a significantly higher resolution was determined than originally reported was also investigated. For this case, one of four reprocessing attempts for the data set reported a resolution higher than that supported by merging statistics. This discrepancy was resolved by a software update.

In addition to estimates of the Bragg intensities, diffraction images can also be analysed for additional features^44^. A well-known example is the isotropic solvent ring that generally appears ∼3–4 Å resolution^45^. However, diffraction images also contain anisotropic diffuse scattering signals under and between the Bragg peaks that derive from two-point correlations of electron density fluctuations^7^. Analysis of this diffuse scattering could therefore provide information about protein, nucleic acid, and lipid structural dynamics and correlated motions, potentially leading to new mechanistic insights^46^ or to validating sampling schemes and energy functions for molecular dynamics simulations^47^. One data set on the model enzyme Cyclophilin A is currently deposited (Table 2) to be used as ‘gold-standard' to compare the influence of temperature on data collection^48^ and to assess consistency between X-Ray Free-Electron Laser (XFEL) and synchrotron data^49^. This data set can now also be analysed for diffuse scattering features, which could distinguish between models of correlated motion suggested by NMR experiments.

### X-ray diffraction reference subset and other collections

To take advantage of data grid diversity, we have selected a small subset of cases that could be used to support software development and teaching of data processing and diverse structure determination techniques (Table 2). This subset includes high-resolution (1.2 Å), low resolution (4.5 and 7.0 Å), anisotropic and twinned data sets. Additionally data sets that supported a variety of experimental phasing approaches (for example, phasing with selenium, zinc, uranium, barium/potassium) and molecular replacement cases (for example, with a 9 Å electron microscopy (EM) envelope) are included. The subset also incorporates diffraction data for crystals grown in lipidic cubic phase and an example of multi-crystal averaging.

Additionally SBDG is suited to support various other primary data types that are being generated by members of the consortium, and those pilot collections will seed development of community-wide data analysis systems. MicroED is a promising new technique^50^,^51^ and inclusions of the early microcrystal data sets might stimulate the community to explore this technique and to fine-tune data processing software. Examples of MicroED data sets that are included in the pilot collection include three MicroED data sets that were used to determine structures of the toxic core of α-synuclein^52^, catalyse^53^ and lysozyme^54^. Other types of data sets in our pilot collection include a 55 GB computational decoy data set for 55 complexes with associated HADDOCK scores^55^, a 2 μs Desmond^56^ MD trajectory^57^, and a recently collected Lattice Light-Sheet Microscopy^58^,^59^ data set with in-vivo imaging of zebrafish embryos^60^. Here the engagement with domain experts and respective communities will be also required to establish data validation pipelines and effective DAA distribution models.

## Discussion

We have developed a flexible data publication system, the SBDG, to support deposition of a variety of large primary data sets. The data repository complements the wwPDB efforts by preserving the raw data that supports PDB-deposited structure models. The pilot phase of the project, which was limited to SBGrid laboratories, demonstrated both feasibility and strong participation, with the deposition and publication of 117 data sets (as of 1 September 2015, collected over 3 months). To support annotated data collection, we have established data processing pipelines that will evolve the post-deposition data-analysis process. For example, the pipeline presented in the results section allows depositors and SBDG curators to quickly identify image-header problems, and parameters that are refined or corrected will be included in the expanded Dataverse schema^61^,^62^,^63^,^64^. The outliers and failures of the current reprocessing pipeline illustrate areas of potential improvement to metadata accuracy and the pipeline itself. Data depositors and other community members will be able to provide data annotations to assist with the convergence of this process. Access to this growing collection of X-ray diffraction data sets will support the proposed paradigm shift in the community^6^ from the static archive towards a much more dynamic body of continuously improving refined models.

Despite being in the age of ‘big data science', universal storage of large, biomedical data sets is an issue that has not yet been resolved, as infrastructure and support responsibilities have not been well defined. Shifting the burdens of data management from individual research groups and institutions to global infrastructures is an effective and economical strategy to address this issue that has previously been proven successful by the wwPDB and would now be demonstrated by the SBDG. By virtue of the consortium's global presence, SBDG is well positioned to stimulate community-wide participation. SBGrid may facilitate integration of the data grid with regional projects and facility-related efforts to preserve primary diffraction data sets. This data distribution model is similar to those established in other fields. For example, the Data Preservation Alliance (www.data-pass.org) replicates and indexes quantitative data for the social sciences. Data collected at the Large Hadron Collider are made available under a multi-tier processing and storage framework. As a large international consortium backed by diverse funding mechanisms and DAA storage contributions of its members, SBGrid is uniquely capable of bypassing grant limitations that would otherwise deter such a long-term global infrastructure effort. Given recently secured funding to support data curation and technology integration under the Dataverse research data management, and with gradual community investment, SBDG is poised to scale up to support the entire community.

While access to experimental data is critical to ensuring research reproducibility, metadata quality is also crucial. Data sets that are poorly annotated have limited use to the research community. With a focus on deployment of a sustainable and flexible data management infrastructure, the SBDG takes a unique approach on metadata preservation. The repository employs an accommodating DataCite schema, which preserves basic information about experiments. The depositions are self-moderated by contributing laboratories, with data publication subject to approval of the principal investigators. As our results demonstrated, this approach worked well for the vast majority of data sets deposited in the SBDG, 82% of which were automatically reprocessed with current data processing software and the majority of the remaining data sets could be easily reprocessed manually. This success rate for reprocessing diffraction data sets was achieved without any explicit quality control to ensure that the data sets contained sufficient information for reprocessing—in other words, using image headers as the only source of experimental (geometry and detector) parameters. Two possibilities under consideration for maintaining and improving this success rate are allowing depositors to annotate updated experimental parameters (for example, beam center) and explicit checks for metadata required for reprocessing prior to data publication. To facilitate interoperability with other projects and further stimulate uniform data evaluations, we will work in parallel to develop tools that will support download of archived data sets in community accepted master formats supporting intrinsic metadata, such as OME-TIFF or HDF5. This process will allow annotation of downloadable data sets with additional information from analysis pipelines, and will be guided by feedback from projects that interface with SBDG. Ideally, publication of data sets will encourage the communities to adopt standardized formats and ensure complete population of experimental metadata with adequate accuracy to support reprocessing.

While the SBDG immediately serves the well-defined area of X-ray crystallography, our pilot project has demonstrated that our infrastructure can preserve additional data types, such as decoy data sets for NMR computations or MicroED data sets. SBDG will duplicate XFEL data sets that are currently accessible through the Coherent X-ray Imaging Data Bank (http://www.cxidb.org/) and support their distribution by DAA. In addition, SBDG will collaborate with MicroED and XFEL collection curators who will moderate development of community driven efforts to automate data analysis pipelines to parallel automatic processing of X-ray diffraction data sets with packages like DIALS or *xia2*. We envision that the tools and technologies that arise from this project will ultimately lead to the development of a fully featured, primary data publication system. Features of such a system would include the capability of supporting a variety of experimental data types and automatic incorporation of pertinent data set information during data collection at local, regional and national facilities. The integration of primary data management with a base set of scientific software enables repositories to progress towards dynamically improving sources of knowledge, as well as providing an integrated computing environment for ongoing research.

In summary, we have presented the SBDG, a new system for the preservation and publication of large experimental data sets. The system is the latest product of SBGrid's mission to maintain a community-wide research-software infrastructure. Through disclosure, adoption, transparency, management of external dependencies, permissible licensing, and technical protection mechanisms, the SBDG is committed to compliance with evolving community standards of data preservation. We expect that the widespread sharing of experimental data will support methods development and will ultimately lead to better quality of structural models that are subject to continuous methods improvement.

## Methods

### Current implementation

The databank deposition process involves five stages: (1) recording associated metadata, (2) local checksum calculation, (3) data transfer, (4) post-transfer verification and (5) public identifier registration.

A publicly accessible web frontend is used for handling user interactions with the databank. Built using the Python-based Django web framework, this frontend runs on an Ubuntu 14 LTS Server with a PostgreSQL 9.3 database. It collects the necessary metadata during deposition and informs the backend systems about deposition requests. A cryptographic checksum (SHA1 SUM, FIPS 180-4) is calculated before data transfer. This ensures that the data set is unchanged. Data transfer is handled by rsync over ssh. Once data transfer is complete, the databank verifies that the data set has been transferred uncorrupted, or reports a problem with the data set. If necessary, extraneous files (intermediate data files, processing or transfer scripts) are removed, data files are uncompressed and checksums re-computed. In the event of any modifications to the data set, an unmodified copy is stored in an offline file system. Upon data set release, the DOI reserved during data set registration is registered using the recorded metadata, and the data set (including checksum information) is made available for download over anonymous rsync.

### Metadata schema

DOIs are issued through EZID, through the Harvard University Library and the California Digital Library. Metadata are organized following the DataCite schema (Fig. 6).

### Reprocessing details

Data sets that had been publicly released by 1 September 2015 were reprocessed by *xia2* in a fully automated manner. For each data set, four attempts were made to reprocess using options ‘-2d', ‘-3d', ‘-3dii' and ‘-dials', using MOSFLM, XDS, XDS (indexing with peaks from all images) and DIALS, respectively. AIMLESS^37^ and POINTLESS^38^ were used by *xia2* for space group determination. A data set was considered successfully reprocessed if any of these attempts succeeded, and comparisons to the originally published structure were done with the best matching result. Investigation of unsuccessfully reprocessed data sets was performed using iMOSFLM^41^. This investigation was performed ‘blinded' to the reprocessing instructions provided by depositors, in order to better investigate the limits of relying solely on diffraction images.

### Data alliance

Released data sets are distributed to Data Alliance mirror sites using the same mechanism as individual data set distribution. Data set checksums enable accurate data transfer. Users can select a mirror site by picking an appropriate rsync URL for data download.

## Additional information

**How to cite this article:** Meyer, P. A. *et al.* Data publication with the structural biology data grid supports live analysis. *Nat. Commun.* 7:10882 doi: 10.1038/ncomms10882 (2016).

## Supplementary Material

Supplementary Information Supplementary Table 1.

## Figures and Tables

**Table 1:** Data science standards.

Disclosure	Software tools developed under this program will be incorporated into open source software and released to the community. Manuscripts and white papers describing various phases of the project will be released on a regular basis.
Adoption	All biomedical image data will be converted to the master formats, such as OME-TIFF or HDF5. Community tools to create, analyse, and manipulate diffraction images will be extended to include support for these formats. All biomedical data are assigned Digital Object Identifiers through the CDL EZID system, and follow modified DataCite and Dataverse metadata schemas. Associated metadata are registered with the International DOI Foundation, making it virtually permanent and independent of SBGrid and Harvard computing infrastructure. All data sets published through the SBDG will be citable using Force 11 recommendations.
Transparency	Files within individual data sets will be deposited in their original format (no archives or encryption allowed). Self-documentation: The majority of diffraction data sets are self-documented and include the basic information required for reprocessing in the images themselves. Additional information will be collected during deposition and will include data set representation (the ability to use the data to be processed), reference (relation to PDB files, publications, and other data sets), context (for example, a native data set or a derivative used for phasing), fixity (checksums), and provenance (typically the data collection facility and the project member who deposits the original data set). With conversion to master formats, all secondary information will be appended to the image metadata along with all original headers.
External dependencies	The ability to reprocess some older data sets and verify master format conversions could depend on access to a specific version of data processing software. As data sets enter our repository, they will be reprocessed with our Data Reprocessing Pipeline (one of several we will develop as part of our Data Mining Pipelines). Data Reprocessing Pipelines will be archived within our system, issued DOIs, and interlinked with the data sets. It is worth noting that, since 2002, SBGrid has been archiving structural biology applications and, therefore, has access to previous software versions that might be required to reprocess older data sets.
Licensing	Biomedical data sets will be deposited under the Creative Commons Zero licence, supporting future development of data validation services and database replications and migrations.
Technical protection mechanism	The security of the deposited data will be maintained by the DAA. The DAA will join with the Library of Congress sponsored NDSA and the data architect working on the project will ensure that NDSA recommendations are being followed.

NDSA, National Digital Stewardship Alliance; SBDG, Structural Biology Data Grid.

**Table 2:** Reference subset.

**Data set**	**Description**
10.15785/SBGRID/5Boggon LaboratoryReference Case 1:MR/Multi-crystal averaging.	Data sets from 5 crystals of SNX17 FERM domain in complex with a peptide corresponding to KRIT1's NPxY2 motif. Separate integration of the data sets and scaling together allows a complete 3.0 Å data set for molecular replacement solution (original paper used 4GXB as a search model) and structure refinement.
	
10.15785/SBGRID/117Baxter LaboratoryReference Case 2:MR/Low resolution, twinned with rotational pseudosymmetry.	3.70 Å data set collected on a crystal of thioester-containing protein 1 *S1 allele (TEP1*S1). Initial data processing suggested P4_3_2_1_2, but one of the two molecules (∼1300 aa. each) in the ASU overlapped with its symmetry-mate. Comparison of alternative scenarios in refinement identified the true space group as *P*4_3_ with twinning and rotational pseudosymmetry. Refinement was completed with TLS, NCS (local) and external restraints derived by *ProSMART*^65^ using TEP1*R1 (PDB 4D94) as reference.
	
10.15785/SBGRID/62Modis LaboratoryReference case 3:U SAD/Low resolution.	4.5 Å data set of a uranyl acetate derivative used for a challenging structure determination by SAD. Certain images had streaky features and were excluded from data reprocessing. The height and definition of peaks in anomalous difference Patterson maps was improved by omitting certain images near the end of the data collection run.
	
10.15785/SBGRID/111 Ferré-D'Amaré LaboratoryReference Case 4:Ba/K SAD; 91 nt RNA-chromophore complex.	2.5 Å data set collected at ALS BL 5.0.2 using 6.0 keV X-rays from a crystal of 'Spinach' a fluorescent RNA analogue of GFP. Although anomalous signal was very weak, a heavy atom substructure comprised of one barium and six potassium ions resulted in good quality SAD electron density maps.
	
10.15785/SBGRID/3Sliz LaboratoryReference Case 5:Zn SAD; 4 Zn/ASUprotein/RNA complex.	2.9 Å Zn SAD data set was sufficient to determine a crystal structure of Lin28/let-7d protein-microRNA complex. X-ray beam size was adjusted to maximize flux and minimize radiation damage. One swapped-dimer is located in each asymmetric unit. Two native zinc atoms are located in each tandem CCHC zinc knuckles domain.
	
10.15785/SBGRID/123Heldwein LaboratoryReference Case 6:3.29-Å SeMet SAD9 Se/ASU	This 3.29-Å selenomethionine SAD data set, collected at 0.9789 Å wavelength at BNL X25 beamline, was sufficient to determine the phases and to trace the structure of HSV-2 gH/gL complex^66^. There are 9 Se sites in the ASU. During integration in HKL2000, χ^2^ appeared very large for some sectors of the data set. These correlated with crystal orientation and likely resulted from a large difference in cell edges (*a*=*b*=88 Å versus *c*=333 Å).
	
10.15785.SBGRID/179Schwartz LaboratoryReference Case 7:MR-SAD at 7.0 Å	Contaminating *E.coli* protein 4FCC_A, acting as a crystallization chaperone, was found readily by MR. Using these MR phases seven (Ta_6_Br_12_)^2+^-positions could be found in the 8.8 Å derivative data set 180. The combined MR-SAD phases were sufficient to position two copies of Nup37 (4FHL) and two copies of Nup120 in the asymmetric unit.
	
10.15785/SBGRID/21810.15785/SBGRID/78Rudenko LaboratoryReference Case 8:MR-SAD at 2.65 Å(44 Se atoms/ASU)	3.25 Å data set (#218) from a crystal of the selenomethionyl neurexin 1alpha ectodomain and 2.65 Å higher resolution native data set (#78), both collected at APS using multiple settings. The structure has 2 molecules/ASU with a total of 14 ordered domains and ∼2,000 residues. Molecular replacement successfully placed 8 LNS domains (using a single LNS domain as a search model, i.e. ∼9% of the scattering mass) generating phases which could be used to reveal 37 out of 44 Se atoms/ASU in the 3.25 Å SeMet SAD data set. Refinement was completed using data set #78.
10.15785/SBGRID/9Tao LaboratoryReference case 9:3.25 Å data set used for MR with a 9-Å cryo-EM envelope	A 3.25-Å resolution data set was collected at APS LS-CAT. The structure was determined by molecular replacement using a 9-Å resolution cryo-EM reconstruction as a phasing model. Solvent flattening and 15-fold noncrystallographic symmetry averaging were applied during phase extension.
	
10.15785/SBGRID/83Drennan LaboratoryReference Case 10:MR/large unit cell, anisotropic.	Diffraction data from different regions of a crystal of Isobutyryl-coenzyme A mutase fused, a 250 kDa dimeric enzyme. This crystal had a large unit cell (*a*,*b*=319 Å, *c*=344 Å) and the data were anisotropic. Separate integration of the 6 wedges with individually adjusted resolution limits and scaling together yields a complete 3.35 Å data set that can be used for molecular replacement.
	
10.15785/SBGRID/125Kruse Laboratory(data collected in Kobilka Laboratory)Reference Case 11:MR, lipidic cubic phase	Diffraction data for lipidic cubic phase crystals of human M_2_ muscarinic acetylcholine receptor bound to the agonist iperoxo, the allosteric modulator LY2119620, and the conformationally-selective nanobody Nb9-8.
	
DOI:10.15785/SBGRID/68Fraser LaboratoryReference case 12:X-ray diffuse scattering	1.2 Å data set collected at SSRL provides a high-resolution standard data set of the enzyme Cyclophilin to examine the influence of data collection temperature to compare with XFEL data, and to measure X-ray diffuse scattering.

MR, molecular replacement; SAD, Single-wavelength Anomalous Diffraction.

12 X-ray diffraction data sets from the SBDG pilot collection were identified as particularly suitable for software testing and teaching activities. In addition, data sets from molecular dynamics, lattice light-sheet microscopy and MicroED represent an invaluable subset.
