# Supplementary material for: Data publication with the structural biology data grid supports live analysis
Source: Nat Commun. 2016 Mar 7;7:10882. doi: 10.1038/ncomms10882 (PMC4786681; doi:10.1038/ncomms10882)
Supplement: Supplementary Information — Supplementary Table 1. [file ncomms10882-s1.pdf]

Principal Investigator	Institution	DOI (10.15785/SBGRID/XXX)	Funding
Anderson, Karen	Yale University School of Medicine	19	NIH GM49551 (to KSA)
Baxter, Richard	Yale University	117,118	NIH GM114358
Blacklow, Stephen	Harvard Medical School	184	Leukemia and Lymphoma Society Specialized Center of Research
Boggon, Titus	Yale University School of Medicine	5	NIH NS085078 (to TJB)
Bonvin, Alexandre	Utrecht University, Bijvoet Center for Biomolecular Research	131	EU e-Infrastructure grants no. 261572 (FP7, WeNMR) and 654142 (H2020, EGI-Engage) and NWO TOP PUNT grant 718.015.001
Brett, Tom	Washington U. School of Medicine	28,29,65,88,89	NIH HL119813 (to TJB)
Caflisch, Amedeo	University of Zurich	93	The Swiss National Science Foundation
Chang, Chung-I	Academia Sinica	87	NSC102-2320-B-001-016 (Ministry of Science and Technology, Taiwan)
Chazin, Walter	Vanderbilt University	66,92	NIH GM065484 and P01 CA092584
Corbett, Kevin	University of California, San Diego	16,17,20,21	NIH GM104141 and the March of Dimes Foundation
Cosgrove, Michael	SUNY Upstate Medical University	18	NIH CA140522 (to MSC)
Crosson, Sean	University of Chicago	116,149,150	NIH GM087353 (to SC)
Dhe-Paganon, Sirano	Dana Farber Cancer Institute	110	Linde Family Program in Cancer Chemical Biology
Di Cera, Enrico	Saint Louis University	70,79	NIH HL49413, HL73813 and HL112303.
Drennan, Catherine	Massachusetts Institute of Technology	83,124	NIH GM069857 (to CLD) and HHMI
Eck, Michael	Dana Farber Cancer Institute	6	American Society of Hematology, NIH CA080942 and the Leukemia and Lymphoma Society (to MJE)
Eichman, Brandt	Vanderbilt University	129,130	NIH ES016486; P30 ES000267; T32 GM08320
Fan, Qing	Columbia University	58,59	NIH GM088454 (to QRF)
Ferre-	National Institutes	111,112	Intramural Program of the

D'Amare, Adrian	of Health		National Heart, Lung and Blood Institute, NIH
Fraser, James	University of California, San Francisco	68	NIH DP5 OD009180 and NSF STC-1231306
Fromme, Chris	Cornell University	75	NIH GM098621 (to JCF)
Garcia, Christopher	Stanford University	69	NIH GM097015, Ludwig Cancer Foundation, and the HHMI
Gaudet, Rachelle	Harvard University	14,15	AHA SDG0335134N and McKnight Scholar Award (to RG)
Gong, Peng	Chinese Academy of Science, Wuhan Branch	119	National Key Basic Research Program of China (2013CB9111)
Harrison, Stephen	Harvard Medical School	24,25,26,27	HHMI
Heldwein, Ekaterina	Tufts University School of Medicine	120,121,122,123,140,141	NIH DP20D001996, NIH R21 AI065886, the Pew Scholar Program in Biomedical Sciences, and Burroughs Wellcome Fund Pathogenesis Award (to EEH)
Jia, Zongchao	Queen's University	73	National Science and Engineering Research Council of Canada (RGPIN 203705-2013)
Keenan, Robert	University of Chicago	113	NIH GM086487 (to RJK)
Kirchhausen, Tom	Harvard Medical School	187	NIH 5R01GM075252
Kruse, Andrew	Harvard Medical School	125	NIH NS028471.
Kvansakul, Marc	La Trobe University	57	NHMRC Career Development Fellowship 637372; NHMRC Project Grant APP1007918 and ARC Future Fellowship FT130101349
McLellan, Jason	Dartmouth Medical School	155,156	NIH 1R43AI112124
Modis, Yorgo	University of Cambridge	61,62	Wellcome Trust 101908/Z/13/Z and R01 GM102869 (to YM)
Otwinowski, Zbyszek	UT Southwestern Medical Center	13	NIH P50GM062414 and NIH U54GM074942
Pai, Emil	University of Toronto	127,128	Canadian Institutes of Health Research (grant # MOP-86548) and the Canadian Research Chairs Program
Pereira, Pedro J.B.	Universidade Do Porto	99-109	PTDC/BIA-PRO/70627/2006 (Fundação para a Ciência e a Tecnologia, Portugal)

Petosa, Carlo	Institut de Biologie Structurale, Grenoble	74,77	ANRS/Fondation de France (AIJC program) and Agence Nationale de la Recherche (NucExp grant)
Raman, C.S.	University of Maryland	85,91,96-97,133-138	NIH AI054444, NIH GM084700, Welch Foundation Grant AU-1574<br>Pew Scholars Program in Biomedical Sciences<br>American Heart Foundation (to CSR)
Rapoport, Tom	Harvard Medical School	80,90	HHMI
Roll-Mecak, Antonina	National Institutes of Health	86	NIH 1ZIANS003122-05
Rosen, Michael	UT Southwestern Medical Center	84, 132	NIH GM056322 and Welch Foundation I-1544 (to MKR), Howard Hughes Medical Institute
Rudenko, Gabrielle	University of Texas Medical Branch	78	NIMH MH077303
Schwartz, Thomas	Massachusetts Institute of Technology	10-12,178-182	NIH R01GM077537, NIH R01AR065484
Shamoo, Yousif	Rice University	71, 72	NIH R01AI080714 (to YS) and Kresge Science Initiative endowment grant
Sliz, Piotr	Harvard Medical School	1-4	NIH CA163647 (to PS)
Sondermann, Holger	Cornell University	94	NIH R01AI097307 (to HS)
Tao, Yizhi (Jane)	Rice University	7,9	Welch Foundation (C-1565 to YJT)
Tolia, Niraj	Washington U. School of Medicine	115	NIH AI080792 and Edward Mallinckrodt, Jr. Foundation (to NHT)
Tsodikov, Oleg	University of Kentucky	22	Igniting Research Collaboration Award from the University of Kentucky
Westover, Ken	UT Southwestern Medical Center	76,157,158-162	CPRIT R1207 and ACS IRG-02-196-07 (to KDW)
Wu, Hao	Boston Children's Hospital	98	NIH AI050872

Supplementary Table 1. Pilot Datasets and Corresponding Grant Support.
